# Quality of life in survivors after a period of hospitalization in the intensive care unit: a systematic review

**DOI:** 10.5935/0103-507X.20180071

**Published:** 2018

**Authors:** Paula Caitano Fontela, Franciele Aline Norberto Branquinho Abdala, Soraia Genebra Ibrahim Forgiarini, Alberto Forgiarini Luiz Jr.

**Affiliations:** 1 Programa de Pós-Graduação em Ciências Pneumológicas, Universidade Federal do Rio Grande do Sul - Porto Alegre (RS), Brasil.; 2 Centro Universitário Metodista IPA - Porto Alegre (RS), Brasil.; 3 Curso de Fisioterapia, Centro Universitário Metodista IPA - Porto Alegre (RS), Brasil.; 4 Programa de Pós-Graduação em Biociências e Reabilitação e Reabilitação e Inclusão, Centro Universitário Metodista IPA - Porto Alegre (RS), Brasil.

**Keywords:** Quality of life, Critical illness, Critical care, Intensive care units, Length of stay

## Abstract

**Objective:**

To assess the long-term, health-related quality of life of intensive care unit survivors by systematic review.

**Methods:**

The search for, and selection and analysis of, observational studies that assessed the health-related quality of life of intensive care unit survivors in the electronic databases LILACS and MEDLINE^®^ (accessed through PubMed) was performed using the indexed MESH terms "quality of life [MeSH Terms]" AND "critically illness [MeSH Terms]". Studies on adult patients without specific prior diseases published in English in the last 5 years were included in this systematic review. The citations were independently selected by three reviewers. Data were standardly and independently retrieved by two reviewers, and the quality of the studies was assessed using the Newcastle-Ottawa scale.

**Results:**

In total, 19 observational cohort and 2 case-control studies of 57,712 critically ill patients were included. The follow-up time of the studies ranged from 6 months to 6 years, and most studies had a 6-month or 1-year follow up. The health-related quality of life was assessed using two generic tools, the EuroQol and the Short Form Health Survey. The overall quality of the studies was low.

**Conclusions:**

Long-term, health-related quality of life is compromised among intensive care unit survivors compared with the corresponding general population. However, it is not significantly affected by the occurrence of sepsis, delirium, and acute kidney injury during intensive care unit admission when compared with that of critically ill patient control groups. High-quality studies are necessary to quantify the health-related quality of life among intensive care unit survivors.

## INTRODUCTION

Technological advances in the intensive care have increasingly reduced intensive care unit (ICU) mortality.^(1)^ However, the consequences of a critical illness can persist for a long time, affecting the physical, cognitive and mental health of ICU survivors.^(2)^The multiplicity of these consequences was recognized as "postintensive care syndrome" and may have a strong, negative impact on functioning and on health-related quality of life (HRQOL).^(2)^

Assessing outcomes related to physical and psychological factors, functional status, social interaction and HRQOL is as important as assessing the long-term mortality rate of ICU survivors.^(3,4)^ Ideally, ICU survivors should reach their premorbidity and/ or admission HRQOL scores or even reach scores that are better than or similar to those of age-, sex- and comorbidity-matched individuals.^(5)^

Although HRQOL scores are increasingly included in studies and recognized as important outcome parameters in this population, such results generate inaccuracy in their interpretation for various reasons. First, the period during which HRQOL recovery should occur in not defined, and therefore, the optimal follow-up period for HRQOL evaluation remains undetermined. Frequently, postintensive care syndrome may clinically manifest as not only transient events,^(6,7)^ occurring 5 years after hospital discharge,^(8)^ but also permanent events, at least for some survivors. Second, baseline HRQOL evaluation is difficult, thus complicating critical illness burden investigations. In addition, the evaluation instruments used in the studies are different, thereby complicating the systematization and interpretation of HRQOL results. However, a better understanding of how much intensive care affects the long-term HRQOL of ICU survivors is necessary to help healthcare professionals in making decisions on future efforts to reduce the burden of critical illness.

The objective of this study was to perform a systematic review, evaluation and synthesis of observational studies published in the literature on the long-term HRQOL of ICU survivors in comparison with the corresponding general population and control groups.

## METHODS

This systematic review was performed according to the Preferred Reporting Items for Systematic Reviews and Meta-Analyses Guidelines (PRISMA). ^(9)^ A systematic search strategy with the indices "*quality of life* [MeSH Terms]" AND "*critically illness* [MeSH Terms]" was used in the electronic databases, Latin American and Caribbean Health Sciences Literature (Literatura Latino-Americana e do caribe em Ciências da Saúde - LILACS) and Medical Literature Analysis and Retrieval System Online (MEDLINE^®^), which were accessed through PubMed from October 13, 2016, to November 7, 2016.

The titles and abstracts of the articles identified in the search strategy were evaluated based on eligibility criteria (Table 1) by three independent reviewers. Full-text articles were downloaded when the abstract lacked information on inclusion and exclusion criteria. The list of references of the selected articles and the personal files of the researchers were also searched for to identify possible studies that might also meet the eligibility criteria of the study and that might not have been found in the initial search. Any discrepancies between the reviewers were resolved by consensus, and a fourth reviewer assessed the publications in cases of persistent disagreement.

**Table 1:** Eligibility criteria for article inclusion and exclusion

Characteristics	Inclusion	Exclusion
Patients	Adult (> 18 years) intensive care unit survivors	Patients with specific diseases or undergoing investigation of certain therapies
Intervention	None	Any
Comparison	General population and/or control group	-
Outcome	Health-related quality of life	-
Study design	Observational study	Randomized, quasi-experimental, qualitative study, case report, review, thesis, editorial, comment
Publication	Language: English; period: last 5 years	-

Data were independently retrieved from the selected articles by two reviewers. Discrepancies were resolved by consensus or by a third reviewer.

The methodological quality of the studies was evaluated using the Newcastle-Ottawa scale by two independent, previously trained and qualified reviewers. The methodological quality score of the cohort and case-control studies was calculated based on three components: study group selection (0 - 4 points), quality of adjustment for confounding factors (0 - 2 points) and evaluation of exposure or outcome of interest (0 - 3 points). The maximum score was 9 points, which expressed high methodological quality. Discrepancies between the reviewers were resolved by consensus, and another evaluation was performed by a third reviewer in case of persistent disagreement.

## RESULTS

### Study selection

**Figure 1:**
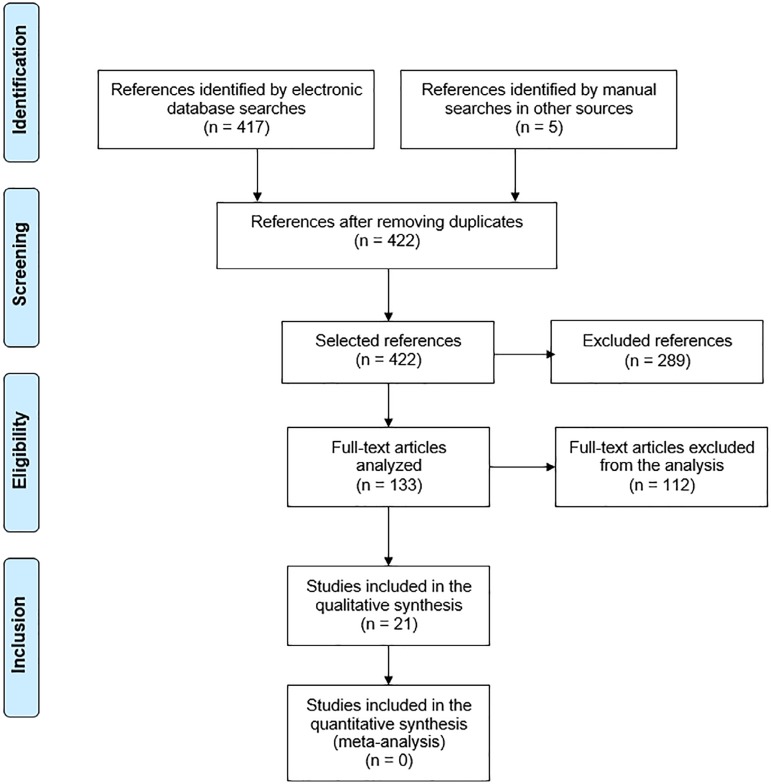
Flowchart of the selection of articles according to the inclusion and exclusion criteria.

The database searches identified 417 references, and the consultation of other sources identified 5 references. Of these references, 289 were excluded because they were published outside of the 5-year period that was stipulated for this review. Of the other references, 112 were excluded after reading the title and the abstract, and eventually the full text, because they failed to meet the other inclusion criteria (Figure 1). There was no discrepancy in the number of articles selected by the three reviewers, and 21 articles were included.

### Characteristics of the included studies

Most studies were conducted in Europe^(10-21)^ and North America,^(22-25)^ followed by Australia,^(26,27)^ China^(28)^ and Argentina.^(29)^ Only one study was conducted in Brazil.^(30)^All studies were observational prospective cohorts, except four, namely, two retrospective cohorts^(12,21)^and two case-control studies.^(28,30)^

The 21 studies included a total of 57,712 critically ill patients, and the individuals were mostly adult, elderly males with varied severity scores on ICU admission. They were classified according to the ICU primary diagnostic groups, such as the following: cardiovascular, respiratory, renal, gastrointestinal, neurological, trauma, orthopedic, surgical, sepsis, hematological, gynecological and metabolic.

### Follow-up time and assessment of health-related quality-of-life

The follow-up times of the studies ranged from 6 months to 6 years, and most studies had a 6-month^(10,12,14,26,27)^ or a 1-year follow-up time.^(13,15,17,21,23-25,29,30)^ HRQOL was assessed using two generic instruments, the EuroQol and the *Medical Outcomes Study 36-Item Short-Form Health Survey (SF-36). The EuroQol version 5D was used in nine studies^(12-14,16,19,21,24,29,30)^ and version 6D in two studies.^(15,17)^ SF-36 was used in 11 studies^(10,11,16,18,19,22,23,25-28)^ and version 12 of the 12-Item Short-Form Health Survey (SF-12) in two studies.^(20,24)^ Long-term HRQOL was evaluated in 24,200 of the 57,712 critically ill patients included in the studies.* The patient characteristics of the studies included in this review and the main findings on long-term HRQOL are outlined in table 2.

**Table 2:** Patient characteristics for the included studies

Reference	Country	Study design	Population	n	Age Mean (SD)	Males n (%)	Disease severity Mean (SD)	ICU diagnosis n (%)	Quality of life
Hofhuis et al.^(10)^	Holland	Prospective 6-month follow-up study	Patients admitted for longer than 48 hours	749	≥ 80 years (n = 129)	72 (56)	APACHE II: 20 (17 - 24)	Cardiovascular: 34 (26) versus 146 (24) Respiratory: 35 (27) versus 211 (34) Gastrointestinal: 55 (43) versus 207 (33) Neurological: 1 (1) versus 29 (5) Trauma: 2 (2) versus 21 (3) Others: 2 (2) versus 6 (1)	Most SF-36 dimensions significantly improved over time. Among the octogenarians, the mean SF-36 dimensions 6 months after ICU discharge were similar to the basal and were not significantly lower than those of the normal population
					< 80 years (n = 620)	385 (62)	APACHE II: 18 (14 - 23)		
van den Boogaard et al.^(11)^	Holland	Prospective 18-month follow-up study	Patients with and without delirium, ICU survivors	915	Patients with delirium: 65 (58 - 75)	101 (60)	APACHE II: 17 (14 - 20)	Surgical: 77 (45) versus 589 (79) Clinical: 54 (32) versus 77 (10) Trauma: 17 (10) versus 24 (3) Neurological: 23 (14) versus 54 (7)	Patients with delirium during the ICU stay had a similar adjusted HRQOL evaluation, albeit with significantly more cognitive problems than patients without delirium
					Patients without delirium: 65 (57 - 72)	508 (68)	APACHE II: 13 (10 - 16)		
Vaara et al.^(12)^	Finland	6-month observational retrospective cohort study	ICU patients treated with RRT or not	24,904	Patients treated with RRT: 63 (52 - 72)	1,143 (67.8)	SAPS II: 48 (37 - 62)	Cardiovascular: 330 (20) versus 6,058 (26) Gastrointestinal: 286 (17) versus 3,628 (16) Neurological: 103 (6) versus 4,483 (19) Renal: 269 (16) versus 383 (2) Respiratory: 159 (9) versus 3,135 (13) Trauma: 36 (2) versus 1,534 (7) Others: 467 (28) versus 3,431 (15)	No clinically significant difference in EQ-5D score was found between patients treated with and without RRT after 6 months of follow up. In addition, a VAS of patients treated with RRT matched the score of patients treated without RRT and that of the general population
					Patients treated without RRT: 62 (50 - 73)	14,641 (63.1)	SOFA: 10 (7 - 13)		
							SAPS II: 33 (23 - 46)		
							SOFA: 6 (3 - 8)		
Pavoni et al.^(13)^	Italy	1-year observational prospective cohort study	Patients aged 80 years or older	288	Clinical: 87 (2)	77 (27)	Clinical SAPS II: 52.3 (8.8)	Clinical: 54 (19)	The HRQOL of clinical and orthopedic elderly patients was worse than the HRQOL of surgical patients and of the normal population 1 year after ICU hospitalization
					Planned abdominal surgery: 87 (1.5)		Planned abdominal surgery (AS): 30.2 (5.4)	Surgical: 74 (26)	
					Unplanned abdominal surgery: 88 (2.2)		Unplanned AS: 46.5 (6.2)	Orthopedic: 160 (55)	
					Orthopedic surgery: 85.9 (4.2)		Orthopedic surgery: 24.2 (7.2)		
Orwelius et al.^(14)^	Portugal	Prospective, multicenter, 6-month follow-up study	Adults ≥ 18 years with ICU stay > 48 hours	313	With community-acquired sepsis: 60 (50 - 70)	59 (65)	SAPS II (IQR): 41 (34 - 51)	Noncoronary: 69 (76) versus 95 (43) Coronary: 1 (1) versus 12 (5) Trauma: 3 (3) versus 46 (21) Elective surgery: 0 (0) versus 35 (16) Nonelective surgery: 18 (20) versus 34 (15)	The long-term HRQOL of patients with community-acquired sepsis were not significantly different compared with ICU patients admitted for other diagnoses. However, when compared with the general population, the HRQOL of patients with sepsis showed a clinically significant decrease
					Without community-acquired sepsis: 59 (43 - 71)	124 (56)	SAPS II (IQR): 35 (27 - 44)		
Wolters et al.^(15)^	Holland	1-year prospective observational cohort study	Patients hospitalized at the ICU for longer than 24 hours	1,101	Patients with delirium: 60.5 (16.7)	271 (66)	APACHE IV: 73.7 (28.3)	Clinical: 222 (54) versus 208 (30) Elective surgery: 96 (23) versus 351 (51) Acute surgery: 94 (23) versus 130 (19)	Delirium during ICU stay is not independently associated with the HRQOL of ICU survivors when adjusted for factors such as severity of illness during ICU stay
					Patients without delirium: 59.4 (16.6)	406 (59)	APACHE IV: 53.9 (22.4)		
Oeyen et al.^(16)^	Belgium	4-year prospective observational cohort study	Patients admitted to the ICU with AKI treated with RRT matched with patients without AKI-TRS	141	Patients with AKI-RRT: 57 (45 - 69)	31 (66)	APACHE II: 26 (21 - 31)	Clinical: 32 (68) versus 67 (71) Elective surgery: 1 (2) versus 4 (4) Emergency surgery: 10 (21) versus 18 (19) Trauma: 3 (6) versus 4 (4) Burn: 1 (2) versus 1 (1)	The long-term QOL of survivors of critical illness with AKI-RRT was similar to that of patients without AKI-RRT, albeit lower than that of the general population
					Patients without AKI-RRT: 57 (48 - 70)	62 (66)	APACHE II: 24 (20 - 30)		
Soliman et al.^(17)^	Holland	1-year prospective cohort study	All ICU patients	5,934	64 (52 - 73)	3.710 (62)	APACHE IV: 49 (35 - 68)	Heart surgery: 2.162 (36) Sepsis: 556 (9) Subarachnoid hemorrhage: 359 (6) Traumatic brain injury: 327 (6) Others: 2.530 (43)	The HRQOL 1 year after ICU hospitalization was significantly lower than that of the sex- and aged-matched general population. However, the 1-year HRQOL markedly varied by ICU survivor subgroup
Hofhius et al.^(18)^	Holland	5-year prospective cohort study	ICU Patients for longer than 48 hours	749	71 (62 - 77)	457 (61)	APACHE II: 19 (14 - 23)	Cardiovascular: 184 (25) Respiratory: 244 (33) Gastrointestinal: 259 (35) Neurological: 30 (4) Trauma: 23 (3) Others: 9 (1)	After correcting for natural decline, the HRQOL significantly decreased, and the physical functioning, social functioning and general health dimensions remained significantly lower than those of the age-matched general population, albeit with small effect sizes
Cuthbertson et al.^(19)^	Scotland	5-year, multicenter, prospective cohort study	Patients with severe sepsis	439	58 (45 - 67)	234 (53)	APACHE II: 23 (17 - 28)	Respiratory: 138 (31) Cardiovascular: 124 (28) Neurological: 44 (10) Gastrointestinal: 93 (21) Other: 26 (7) Unknown: 14 (3)	Patients with severe sepsis have a significantly lower physical dimension of HRQOL than the normal population, although the mental dimension was slightly lower than the normative data up to 5 years after severe sepsis
							SAPS II: 41 (30 - 54)		
Battle et al.^(20)^	United Kingdom	2-year, observational, prospective cohort study	Patients with sepsis	50 Group SIRS: (n = 19)	58 (30)	23 (46)	SOFA: 3 (4)	Respiratory: 18 (36) Gastrointestinal: 9 (18) Neurological: 1 (2) Endocrine: 10 (20) Renal: 8 (16) Others: 4 (8)	The HRQOL of patients with sepsis was significantly lower than local, normative data. More significant decreases in HRQOL were found in patients with septic shock than in patients with SIRS and sepsis
				Group sepsis: (n = 16)			Charlson Comorbidity Index: 3 (4)		
				Septic shock group: (n = 15)					
Honselmann et al.^(21)^	Germany	1-year retrospective cohort study	Patients with pneumonia and/or sepsis	217	71 (62 - 78)	134 (62)	SAPS II: 36 (28 - 50)	Sepsis: 145 (67) Pneumonia: 72 (33) Sepsis and pneumonia: 99 (46)	The HRQOL of patients with pneumonia and/or sepsis was significantly lower than that of the local reference group
Fan et al.^(22)^	United States	2-year, multisite, prospective study with longitudinal follow-up	Patients under MV with ALI	222	49 (40 - 58)	123 (55)	APACHE II: 23 (19 - 28)	Pneumonia: 112 (50) Sepsis: 44 (20) Aspiration: 29 (13) Trauma: 7 (3) Others: 30 (14)	The physical function was substantially impaired, when compared with the corresponding population in all time points (3, 6, 12 and 24 months), and remained markedly impaired in relation to baseline values estimated before ALI (72% baseline value at 24 months of follow-up), according to the SF-36 questionnaire
Heyland et al.^(23)^	Canada	1-year multicenter, prospective, observational cohort study	Patients aged 80 years or older admitted to the ICU for less than 24 hours	610	84 (80 - 99)	338 (55)	APACHE II: 22 (7 - 49)	Cardiovascular: 94 (15) Respiratory: 94 (15) Sepsis: 135 (22) Gastrointestinal: 110 (18) CVA: 27 (4) Neurological: 20 (3) Trauma: 46 (8) Metabolic: 8 (1) Hematological: 18 (3) ABG/ valve replacement: 49 (8) Renal: 2 (0) Gynecological: 1 (0) Orthopedic: 6 (1)	Octogenarian ICU survivors had significantly lower SF-36 scores in the physical functioning section than those of sex- and age-matched controls in all time points (3, 6, 9 and 12 months). A quarter of them returned to baseline levels of physical functioning at 12 months
Bagshaw et al.^(24)^	Canada	1-year multicenter, prospective, observational cohort study	ICU patients aged 50 years or older for more than 24 hours	421	Frail: 69 (10.1)	72 (52)	APACHE II 21.3 (6.5)	Surgery: 34 (26) versus 108 (38) Cardiac arrest: 10 (7) versus 21 (7) Mechanical ventilation: 122 (88) versus 240 (85) Vasoactive drug therapy: 83 (60) versus 146 (52) Renal replacement therapy: 14 (10) versus 33 (12)	12 months after critical illness, frail patients had a worse HRQOL, both in the EuroQol and in the SF-12 , than nonfrail survivors and the general population
					Not frail: 66.2 (9.7)	186 (66)	APACHE II 18.6 (7.1)		
Feemster et al.^(25)^	United States	1-year prospective, observational cohort study	All patients who visited their primary care unit at least once in the previous year	11,243	Outpatient: 64.8 (10.8)	8.929 (97)	-		Hospital and ICU patients showed clinically significant decreases in SF-36 sections, albeit small and similar between both groups
					Hospital patient: 66.0 (10.7)	1.297 (96)			
					ICU patient: 66.8 (9.4)	649 (97)			
McKinley et al.^(26)^	Australia	6-month prospective, observational cohort study	Adult patients subjected to MV for longer than 24 hours and who stayed at the ICU for longer than 48 hours	195	57 (16)	116 (59)	APACHE II: 18.8 (6.9)	Cardiovascular: 38 (20) Respiratory: 46 (24) Gastrointestinal: 57 (30) Others: 50 (26)	The mental health score of the SF-36 at 1 week was lower than the mean score of the age-matched population, although it improved within 8 to 26 weeks after hospital discharge
McKinley et al.^(27)^	Australia	6-month prospective, observational cohort study	Adult ICU patients for longer than 48 hours	222	57.2 (17.2)	145 (65)	-	Cardiovascular: 81 (37) Respiratory: 13 (6) Gastrointestinal: 20 (9) Neurological: 57 (26) Trauma: 24 (11) Sepsis: 5 (2) Others: 22 (10)	The mental health and physical functioning scores of the SF-36 were significantly lower in patients with poor sleep quality 6 months after ICU discharge
Zhang K et al.^(28)^	China	6-year, multicenter, prospective case-control study	Adults, ≥ 18 years, who stayed at the ICU for longer than 24 hours	224	Sepsis group: 53 (17.3)	Sepsis group: 32 (76)	APACHE II: 18.3 (6.8)	Cardiovascular: 14 (33) versus 9 (27) Respiratory: 22 (52) versus 12 (36) Renal: 12 (28) versus 9 (27) Hematological: 9 (21) versus 5 (15) Neurological: 23 (54) versus 16 (48)	No difference in HRQOL was found between the sepsis and the control groups of critically ill patients. However, when compared the community control group, the patients with severe sepsis showed clinically significant impairment in 4 of the 8 domains of the SF-36 6 years after hospital discharge
					Control group: 47 (18.1)	Control group: 23 (70)	APACHE II: 13.7 (6.5)		
Das Neves et al.^(29)^	Argentina	1-year, prospective, observational cohort study	Patients aged 15 years or older who remained under MV for longer than 48 hours	112	33 (24 - 49)	76 (68)	APACHE II: 15 (6)	Trauma: 56 (50) Traumatic brain injury: 46 (41) Medical: 32 (29) Emergency surgery: 15 (13) Elective Surgery: 9 (8)	The patients showed high and persistent critical illness burden, severely affecting their HRQOL, which was adversely affected by events such as shock, MV duration and persistent weakness
Contrin et al.^(30)^	Brazil	1-year, nested case-control study	Patients with severe sepsis	100	Control group: 52.2 (19.4)	24 (48)	_	Respiratory: 4 (8) versus 11 (22) Urinary: 3 (6) versus 5 (10) Cardiovascular: 3 (6) versus 4 (8) Nervous: 9 (18) versus 5 (10) Trauma: 6 (12) versus 10 (20) Gastrointestinal: 12 (24) versus 4 (8) Neoplasia: 10 (20) versus 4 (8) Sepsis: 2 (4) versus 1 (2) Metabolic: 0 (0) versus 2 (4) Postoperative: 1 (2) versus 4 (8)	Elderly patients with sepsis had more moderate and severe problems in all five dimensions of the HRQOL studied than critically ill patients without sepsis
					Sepsis group: 51.3 (20.0)	32 (64)	_		

SD – standard deviation; ICU – intensive care unit; APACHE – Acute Physiology and Chronic Health Evaluation; SF-36 – Medical Outcomes Study 36 - Item Short - Form Health Survey; HRQOL – health-related quality of life; RRT – renal replacement therapy; SAPS – Acute Physiology Score; SOFA – Sequential Organ Failure Assessment; EQ-5D – EuroQol Health Questionnaire; VAS – visual analog scale; IQR – interquartile range; AKI – acute kidney injury; QOL – quality of life; SIRS – systemic inflammatory response syndrome; MV – mechanical ventilation; ALI – acute lung injury; CVA – cerebrovascular accident (stroke); SF-12 – 12-Item Short-Form Health Survey.

In 18 of the 21 studies included in this review, the long-term HRQOL of ICU survivors was compromised when compared with that of the corresponding general population. In studies with a 6-month follow-up time, the HRQOL of critically ill and elderly patients with low severity scores and critically ill patients with acute kidney injury was similar to that of the corresponding general population.^(10,12,26)^Most HRQOL dimensions improved in the long run.^(10,16,18,19,22,24,26,29)^The domains related to physical aspects were the most affected.^(13,18,19,22,23,27)^

### Risk of bias in the included studies

The general methodological quality of the studies included in this review was low (Table 3). The Newcastle-Ottawa scores of the studies ranged from 2 to 5; a score lower than 4 indicated limited or low-quality evidence. Consensus was reached on all occasions, and no study was excluded from this review, based on the risk of bias assessed. Meta-analyses could not be performed because the studies included in this review had a predominantly observational cohort design.

**Table 3:** Risk of bias for cohort and case-control studies using the Newcastle-Ottawa scale

Reference	Design	Selection	Comparability	Outcome	Total
Hofhuis et al.^(10)^	Prospective cohort study	2	0	3	5
Van den Boogaard et al.^(11)^	Prospective cohort study	2	0	2	4
Vaara et al.^(12)^	Retrospective cohort study	2	0	2	4
Pavoni et al.^(13)^	Prospective cohort study	2	0	2	4
Orwelius et al.^(14)^	Prospective cohort study	2	0	2	4
Wolters et al.^(15)^	Prospective cohort study	1	0	1	2
Oeyen et al.^(16)^	Prospective cohort study	2	1	2	5
Soliman et al.^(17)^	Prospective cohort study	1	0	1	2
Hofhuis et al.^(18)^	Prospective cohort study	2	0	2	4
Cuthbertson et al.^(19)^	Prospective cohort study	2	1	1	4
Battle et al.^(20)^	Prospective cohort study	1	0	1	2
Honselmann et al.^(21)^	Retrospective cohort study	1	1	2	4
Fan et al.^(22^	Prospective cohort study	1	1	2	4
Heyland et al.^(23)^	Prospective cohort study	2	0	2	4
Bagshaw et al.^(24)^	Prospective cohort study	1	0	2	3
Feemster et al.^(25)^	Prospective cohort study	2	0	2	4
McKinley et al.^(26)^	Prospective cohort study	1	0	2	3
McKinley et al.^(27)^	Prospective cohort study	2	1	2	5
Zhang K et al.^(28)^	Prospective case-control study	2	1	1	4
Das Neves et al.^(29)^	Prospective cohort study	1	0	2	3
Contrin et al.^(30)^	Nested case-control study	2	0	2	4

Strong evidence – 6/9 consistent findings among several high-quality studies; moderately strong evidence – 4-5/9 consistent findings among several low- and/ or high-quality studies; Limited evidence – < 4 low-quality studies; conflicting evidence – inconsistent findings among multiple studies; no evidence – no evidence among studies.

## DISCUSSION

This systematic review describes the long-term HRQOL of ICU survivors. In total, 21 studies were included in this review. The overall quality of the studies was low, according to the Newcastle-Ottawa scale, thus highlighting the need for studies with high methodological quality to determine the long-term HRQOL of ICU survivors. New studies with appropriate methodological designs may provide important data on the main factors that result in their change, as well as on possible therapeutic alternatives.

### Critically ill patients

The long-term HRQOL of the critically ill patients differed among the studies analyzed, varying by population and follow-up time. In the short term, the mental component of the HRQOL in a population of critically ill patients with a low severity score was similar to that of the corresponding age-matched population at 8 and 26 weeks after hospital discharge.^(26)^ In a 1-year follow-up, three studies, which were conducted in Holland,^(17)^ Argentina^(29)^ and the United States,^(25)^ highlighted that HRQOL was significantly compromised and that it was even more affected among specific subgroups of ICU survivors, such as those diagnosed with shock who remained under mechanical ventilation for a long period and who showed persistent weakness.^(17,29)^Conversely, a study that compared the impact on HRQOL between hospital and ICU patients highlighted that HRQOL is clinically impaired in both groups 1 year after discharge, with no significant difference between hospital and ICU patients.^(25)^

In the 5-year period, after correcting for natural decline, the HRQOL of ICU survivors significantly decreased, and the physical and social functioning and overall health domains of ICU survivors remained significantly lower than those of the age-matched general population.^(18)^ However, the size effect of the HRQOL reduction was weak on all domains of the evaluation instrument, thus suggesting that the ICU admission effect on the perception of HRQOL 5 years after discharge may not be clinically relevant.

### Elderly critically ill patients

Among elderly ICU survivors, the HRQOL 1 year after ICU discharge was worse than that of the age-^(13)^ and age- and sex-matched^(23)^ general population. Conversely, in another study^(10)^ with a shorter follow-up time, which was performed in an elderly population with lower severity scores, the HRQOL scores 6 months after ICU discharge were similar both to the scores before ICU admission, which were obtained by a patient representative, and to those of the age-matched general population. In both studies^(13,23)^ that showed impaired HRQOL, physical functioning was the most affected domain. Understandably, elderly ICU survivors show impaired HRQOL given their likely increase in comorbidities, and this impairment was more visible in physical functioning.

### Critically ill patients with sepsis

The HRQOL of critically ill patients with sepsis was not significantly different from that of nonelderly critically ill patients with sepsis,^(30)^ of critically ill patients with community-acquired sepsis^(14)^or of age-, sex- and Charlson comorbidity index-matched critically ill patients without sepsis,^(28)^ both in 6-month^(14)^ and in 1-^(30)^ and 6-year follow-up studies.^(28)^ However, the HRQOL of these patients showed a clinically relevant impairment when compared with the general population.^(14,19-21,28)^ Approximately 50 to 75% patients with sepsis^(7)^ progressed with ICU-acquired muscle weakness, which is one of the main signs of physical function impairment of postintensive care syndrome, versus 25 to 50% patients subjected to mechanical ventilation.^(7)^ However, the short-^(14)^ and long-term^(28,30)^ HRQOL of these patients was not significantly different in comparison with critically ill patients with other diagnoses, thus showing that ICU admission, regardless of diagnosis and patient clinical status, is the determinant of impaired HRQOL in these survivors.

### Critically ill patients with delirium

The HRQOL of patients with *delirium* during their ICU stay was not significantly affected compared with patients without delirium after 12^(15)^ and 18 months of follow-up,^(11)^although they showed significantly more cognitive problems.^(11)^ Previous studies^(31,32)^ have shown that *delirium* during an ICU stay is associated with long-term cognitive deficit and mortality, leading to speculation that delirium would also affect the long-term HRQOL, which has not been fully elucidated yet.

### Critically ill patients with acute kidney injury treated with renal replacement therapy

Two studies^(12,16)^investigated the HRQOL of critically ill patients with acute kidney injury treated with renal replacement therapy. Both studies found no long-term HRQOL differences between critically ill patients with acute kidney injury and those without. However, the studies differed when comparing the HRQOL of critically ill patients with acute kidney injury with the HRQOL of the healthy population. Vaara et al.^(12)^ found no differences at 6 months, whereas Oeyen et al.^(16)^ found significant differences after 4 years of follow-up. Importantly, Vaara et al.^(12)^ conducted a retrospective short-term study.

### Critically ill patients with acute lung injury

Only one study^(22)^ evaluated the HRQOL of critically ill patients with acute lung injury. In this population, which was evaluated in the United States, the baseline values of the physical functioning component of the HRQOL were substantially lower than those estimated at 2 years of follow-up. A previous and highly relevant study^(8)^ on the subject demonstrated that ICU survivors who developed acute respiratory distress syndrome showed physical and psychological sequelae and a reduced physical function component of the HRQOL 5 years after ICU discharge, corroborating the finding of the study included in this review.

### Critically ill patients with poor sleep quality

In individuals with poor sleep quality, the only study published on HRQOL in this population demonstrated that the physical and mental functioning components of the evaluation instrument were significantly lower in these individuals 6 months after ICU discharge.^(27)^ Evidence indicates that low quality of ICU sleep and acute sleep deprivation lead to possible negative effects on recovery in critically ill patients,^(33,34)^ including physical and psychological recovery.

## CRITICALLY ILL PATIENTS WITH FRAILTY

Frailty is a multidimensional state characterized by physiological and cognitive loss in older patients, and it predicts adverse events and unfavorable outcomes.^(35)^ Critically ill patients in a state of frailty classified with the Canadian Study of Health and Ageing Clinical Frailty Scale were evaluated in a multicenter cohort in Canada 1 year after ICU admission.^(24)^ These individuals showed worse HRQOL scores than nonfrail individuals and the healthy population.^(24)^ Another study included in this review, performed with elderly people aged 80 years or older, demonstrated that frailty was an independent predictor that was more significant than age, critical illness severity or comorbidities - which are commonly considered key determinants of long-term outcome.^(23)^

### Postintensive care syndrome

A substantial, albeit unknown, proportion of ICU survivors is at risk of developing postintensive care syndrome. Increasing efforts have been made to use the term "postintensive care syndrome" to describe new or aggravated physical, cognitive or mental deficits, resulting from critical illness, that persist after hospitalization,^(2,36)^ with the aim of understanding the epidemiology of this syndrome and its burden on the long-term HRQOL of ICU survivors. Approximately 25 to 50% of patients subjected to mechanical ventilation will develop ICU-acquired muscle weakness,^(7)^ and approximately 85 to 95% of them persist with neuromuscular deficits for 2 to 5 years after hospital discharge.^(7)^ Approximately 30 to 80% patients show cognitive impairment after their ICU stay,^(37)^ and 10 to 50% patients show new depression, anxiety, posttraumatic stress and sleep disorder symptoms.^(7)^ The high and persistent prevalence of changes related to postintensive care syndrome apparently justifies the long-term negative effects on the HRQOL of ICU survivors, and these consequences are more prominent in some specific situations found in the intensive care setting, as shown in this systematic review.

Importantly, the long-term HRQOL assessment presented by these studies clearly disregarded patients who dropped out or died, as only 42% (24,200 patients) of the total sample (57,712) was assessed. We believe that this finding compromises the HRQOL assessment of this population because sample loss may be related to worsened patient clinical status or death.

The search strategy used in electronic databases failed to identify some eligible studies. Previous systematic reviews^(38,39)^ on the subject used broader search strategies such as the following: ("*quality of life*" *OR* "*health status indicators*") *AND* ("*intensive care units*" *OR* "*critical care*" *OR* "*critical illness*" *OR* "*sepsis*" *OR* "adult respiratory distress syndrome") and ("*quality of life*" *OR* "*long-term outcome*") *AND* ("*intensive care*" *OR* "*critical care*" *OR* "*critically ill patients*" *OR* "*ICU patients*" *OR* "*critical care patients*" *OR* "*ICU stay*" *OR* "*ICU*"). Dowdy et al.^(38)^ included the terms "sepsis" and "acute respiratory distress syndrome" in the search strategy because an eligible study identified before conducting the search was not identified when using the initial terms. However, we complemented the search in the reference lists of other systematic reviews and relevant publications on the subject.

The scope of this review was comprehensive; therefore, the heterogeneity of the studies was a limitation, precluding their comparison. We chose to broaden our review to better describe the potential burden of ICU hospitalization on long-term HRQOL. Future, high-quality studies in specific populations are necessary to prepare meta-analyses for specific ICU groups.

## FINAL CONSIDERATIONS

Long-term, health-related quality of life is compromised among intensive care unit survivors when compared with the corresponding general population. However, long-term, health-related quality of life is not significantly affected by the presence of sepsis, delirium or acute kidney injury during intensive care unit stay when compared with that of critically ill patient control groups. High-quality studies are necessary to quantify the health-related quality of life of intensive care unit survivors.
